# Prevention of Syphilis and Cutaueous Diseases in the French Army

**Published:** 1843-01

**Authors:** 


### Prevention of Syphilis and Cutaneous Diseases in the French Army.

A measure has been recently adopted for this purpose. Hitherto every venereal soldier on leaving the hospital was punished by a month's arrest of pay (*consigne*). The consequence was that the soldiers concealed the disease as long as possible, and resorted to quacks to be treated: bad cases therefore often occurred, and the cures were long and expensive. The punishment is now abolished for soldiers who voluntarily confess their disease on the appearance of the first symptoms of syphilis or itch; but they are still amenable to it if the appearance of primary symptoms has existed more than four days, and it is so distinct that they could not have mistaken it. Another new arrangement admits soldiers who have been absent on a week's leave or more, or who belong to the reserve, and are attacked by venereal diseases or the itch, to be treated at the expense of the state in the civil or military hospitals, provided they present themselves at the commencement of the affection.

*Bulletin Génerale de Thèrapeutique. Juin*, 1842.

